# Preventive effects of the angiotensin-converting enzyme inhibitor, captopril, on the development of azoxymethane-induced colonic preneoplastic lesions in diabetic and hypertensive rats

**DOI:** 10.3892/ol.2014.2136

**Published:** 2014-05-12

**Authors:** TAKAHIRO KOCHI, MASAHITO SHIMIZU, TOMOHIKO OHNO, ATSUSHI BABA, TAKAFUMI SUMI, MASAYA KUBOTA, YOHEI SHIRAKAMI, HISASHI TSURUMI, TAKUJI TANAKA, HISATAKA MORIWAKI

**Affiliations:** 1 Department of Medicine/Gastroenterology, Gifu University Graduate School of Medicine, Gifu, Chūbu 501-1194, Japan; 2 Department of Tumor Pathology, Gifu University Graduate School of Medicine, Gifu, Chūbu 501-1194, Japan

**Keywords:** metabolic syndrome, colon carcinogenesis, hypertension, angiotensin-converting enzyme inhibitor, renin-angiotensin system

## Abstract

Metabolic syndrome (Mets), including diabetes and hypertension, increases the risk of colorectal cancer via the induction of chronic inflammation, acceleration of oxidative stress, and activation of the renin-angiotensin system. The present study examined the possible inhibitory effects of captopril, an angiotensin-converting enzyme (ACE) inhibitor and antihypertensive drug, on the development of azoxymethane (AOM)-induced colonic premalignant lesions, aberrant crypt foci (ACF), in SHRSP.Z-*Lepr^fa^*/IzmDmcr (SHRSP-ZF) diabetic and hypertensive rats. Male 6-week-old SHRSP-ZF rats were administered two, weekly intraperitoneal injections of AOM (20 mg/kg body weight). Following the second injection, the rats received drinking water containing captopril (8 mg/kg/day) for two weeks. At sacrifice, captopril administration significantly lowered the blood pressure and reduced the total number and size of ACF compared with those observed in the untreated group. The serum levels of angiotensin-II and the expression levels of *ACE* and angiotensin-II type 1 receptor mRNA on the colonic mucosa decreased following captopril treatment. Captopril also reduced the urinary 8-hydroxy-2′-deoxyguanosine levels and the serum derivatives of reactive oxygen metabolites levels, both of which are oxidative stress markers, but increased the mRNA levels of catalase, an antioxidant enzyme, in the colonic epithelium. Moreover, the expression levels of tumor necrosis factor-α, interleukin-18, monocyte chemoattractant protein-1, inducible nitric oxide synthase, vascular endothelial growth factor and proliferating cell nuclear antigen mRNA in the colonic epithelium were decreased significantly following captopril administration. These observations suggested that captopril prevents the development of ACF by inhibiting renin-angiotensin system activation and attenuating inflammation and oxidative stress in SHRSP-ZF rats. Therefore, targeting Mets-related pathophysiological conditions, including renin-angiotensin system activation, may be an effective strategy to prevent colorectal carcinogenesis in patients with Mets, particularly those with hypertension.

## Introduction

Colorectal cancer (CRC) is a serious health problem worldwide and recent evidence indicates that obesity and metabolic syndrome (Mets), both of which are also global health problems, closely correlate with an increased risk of CRC development (1–5). Several pathophysiological mechanisms, such as the emergence of insulin resistance, state of chronic inflammation and the induction of oxidative stress, may be involved in colorectal carcinogenesis in patients with Mets (3–5). For example, diabetic patients, who frequently present with Mets in addition to insulin resistance, are considered as a high-risk group for CRC development (1–5). However, several rodent studies have demonstrated that targeting insulin resistance and chronic inflammation is effective for preventing obesity- and diabetes-related colorectal carcinogenesis (4,6–9).

In addition to diabetes, epidemiological studies have revealed that hypertension, which is an additional component of Mets, may increase the risk of CRC (1,2). Hypertension is critically involved in the early stage of colorectal carcinogenesis via the activation of the renin-angiotensin system and subsequent induction of oxidative stress and chronic inflammation (10). The renin-angiotensin system is important in the regulation of blood pressure and hydromineral balance, and its activation is one of the key factors in the etiology of Mets, particularly hypertension (11). Angiotensin-converting enzyme (ACE) cleaves angiotensin (AT)-I to AT-II, which is the active product of the renin-angiotensin system and exerts a physiological effect through binding to its receptor, AT-II type 1 receptor (AT-1R) (12,13). Therefore, the renin-angiotensin system inhibitors, including ACE inhibitors and AT-1R blockers (ARB), are used widely for the treatment of hypertension. Furthermore, ACE inhibitors have been shown to exert beneficial effects on cardiovascular disease and reduce mortality as a result of hypertension (14,15).

In addition to the regulation of cardiovascular function, the renin-angiotensin system, which exists in multiple tissues, including the colon, exhibit functions in effecting tissue angiogenesis and chronic inflammation, as well as controlling cellular proliferation and apoptosis. Furthermore, abnormalities in the renin-angiotensin system closely correlate with the enhancement of cancer cell migration, invasion and metastasis, which are correlated with poor prognosis (16–18). The levels of gene expression and enzymatic activity of ACE are increased in human colon adenocarcinoma tissues (19). These aforementioned studies indicate that dysregulation of the renin-angiotensin system may be significant in Mets-related colorectal carcinogenesis and, therefore, an effective target for the chemoprevention of CRC, specifically in patients with Mets.

SHRSP.Z-*Lepr^fa^*/IzmDmcr (SHRSP-ZF) rats were established as a new model of human Mets by crossing SHRSP rats, which have a higher blood pressure, with obese and diabetic Zucker fatty rats (20,21). In our previous study, a new Mets-related colorectal carcinogenesis model was established using SHRSP-ZF rats and a colonic carcinogen, azoxymethane (AOM) (10). In this model, the activation of the renin-angiotensin system and subsequent augmentation of chronic inflammation and oxidative stress enhanced the development of AOM-induced colonic premalignant lesions, aberrant crypt foci (ACF), which indicated that the model was useful to test the potential efficacy of renin-angiotensin system inhibitors in preventing CRC development in patients with Mets (10). The objective of the present study was to examine the preventive effects of captopril, a widely used ACE inhibitor in hypertensive patients, on the development of AOM-induced ACF in diabetic and hypertensive SHRSP-ZF rats.

## Materials and methods

### Animals and chemicals

Five-week-old male SHRSP-ZF rats (n=20) were obtained from the Japan SLC (Shizuoka, Japan) and humanely maintained at Gifu University Life Science Research Center (Gifu, Japan) in accordance with the Institutional Animal Care Guidelines. AOM was purchased from Wako Pure Chemical Industries, Ltd. (Osaka, Japan) and captopril was obtained from Sigma-Aldrich (St. Louis, MO, USA). The study was approved by the ethics committee of Gifu University Life Science Research Center (Gifu, Japan).

### Experimental procedure

After one week of acclimatization, the rats were separated into two groups of 10 rats each. All rats received an intraperitoneal injection of AOM (20 mg/kg body weight) once a week for two weeks. One week following the second injection of AOM, the rats were administered water with or without captopril (8 mg/kg/day) for two weeks. The intake of captopril was maintained by adjusting its concentration in the drinking water, the volume of which was measured three times a week (22). At the end of the experiment, when the rats were 10 weeks of age, systolic and diastolic blood pressures were measured non-invasively using a tail cuff (Softron BP98A; Softron, Tokyo, Japan) and all rats were sacrificed by CO_2_ asphyxiation for colon resection. The third portion of the excised colons (cecum side) was used to extract RNA, and the remaining portion was used to determine the number of ACF (10,23).

### Number of ACF

The frequency of ACF was determined as previously described (10,23). The colon samples were fixed with 10% buffered formalin, stained with methylene blue (0.5% in distilled water; Wako Pure Chemical Industries, Ltd.) for 20 sec and then placed on microscope slides to count the number of ACF using a BH2 Olympus microscope (Olympus, Tokyo, Japan). The number of ACF was recorded along with the number of aberrant crypts (ACs) in each focus. Data are presented as per unit area (cm^2^).

### RNA extraction and quantitative polymerase chain reaction (qPCR)

The isolation of epithelial crypts, extraction of total RNA from isolated epithelial crypts, amplification of cDNA from total RNA and qPCR analysis were performed as previously described (10,23). The sequences of specific primers that amplify tumor necrosis factor α (*TNF-α)*, interleukin 18 (*IL-18*), monocyte chemoattractant protein 1 (*MCP-1*), inducible nitric oxide synthase (*iNOS*), *ACE*, *AT-1R*, vascular endothelial growth factor (*VEGF*), catalase (*CAT*), proliferating cell nuclear antigen (*PCNA*) and glyceraldehyde-3-phosphate dehydrogenase (*GAPDH*) genes were obtained from Primer-BLAST (http://www.ncbi.nlm.nih.gov/tools/primer-blast/; Table I). Each sample was analyzed on a LightCycler Nano (Roche Diagnostics, Basel, Switzerland) with FastStart Essential DNA Green Master (Roche Diagnostics). Parallel amplification of *GAPDH* was used as the internal control.

### Clinical chemistry

The blood samples, which were collected at the time of sacrifice after 6 h of fasting, were used for chemical analyses. The serum levels of insulin (Shibayagi, Gunma, Japan), glucose (BioVision Research Products, Mountain View, CA, USA), leptin (Shibayagi), triglyceride (Wako Pure Chemical Industries, Ltd.), non-esterified fatty acid (NEFA; Wako Pure Chemical Industries, Ltd.) and AT-II (Phoenix Pharmaceuticals, Inc., Burlingame, CA, USA) were determined by an enzyme-linked immunosorbent assay (ELISA) kit according to the manufacturer’s protocols (NIKKEN SEIL Co. Ltd., Shizuoka, Japan).

### Oxidative stress analysis

Urine 8-hydroxy-2′-deoxyguanosine (8-OHdG) levels were determined using the ELISA kit (NIKKEN SEIL Co. Ltd.). Serum levels of hydroperoxide, a marker for oxidative stress, were evaluated using the derivatives of reactive oxygen metabolites (d-ROM) test (FREE Carpe Diem; Diacron International s.r.l., Grosseto, Italy) (10,24).

### Statistical analysis

All data are presented as the mean ± standard deviation and were analyzed using JMP 9 (Statistical Analysis System Institute, Inc., Cary, NC, USA) for Windows. Student’s t-test was performed to compare the mean values among the groups. P<0.05 was considered to indicate a statistically significant difference.

## Results

### General observations

Irrespective of captopril administration, no significant differences were observed in the mean body weights of experimental rats at the termination of the experiment (10 weeks of age; Table II). The mean adipose tissue weights increased in the rats treated with captopril (P<0.05) and treatment with captopril effectively lowered the systolic and diastolic blood pressures (P<0.05). Histopathological examinations of the liver, kidney and spleen confirmed the absence of toxicity from captopril (data not shown).

### Effects of captopril on AOM-induced ACF and colonic epithelial expression of PCNA mRNA in SHRSP-ZF rats

**Figure 1:**
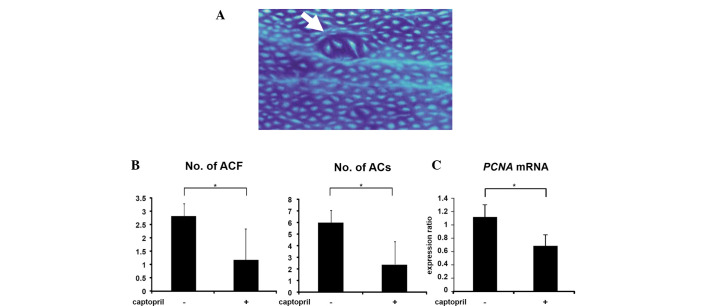
Effects of captopril on AOM-induced ACF formation and colonic epithelial expression of *PCNA* mRNA in the experimental rats. (A) Representative morphology of ACF (as indicated by the arrow) induced by AOM stained with methylene blue in captopril-untreated rats (magnification, ×40). (B) Average number of ACF and ACs (/cm^2^) in captopril-untreated and captopril-treated groups. (C) The expression levels of *PCNA* mRNA in the colonic epithelium were examined by quantitative polymerase chain reaction using specific primers. Data are presented as the mean ± standard deviation. ^*^P<0.05. AOM, azoxymethane; ACF, aberrant crypt foci; PCNA, proliferating cell nuclear antigen; ACs, aberrant crypts.

At the end of the study, ACF (Fig. 1A) were observed in the colons of all rats that received AOM. However, captopril treatment significantly reduced the number and size (ACs per cm^2^) of the ACF in these rats (Fig. 1B; P<0.05). In addition, the colonic epithelial expression of *PCNA* mRNA decreased significantly with captopril administration (Fig. 1C; P<0.05). These observations suggested that captopril inhibits the early stage of colorectal carcinogenesis in obese and hypertensive rats, at least in part, through the suppression of cell proliferation.

### Effects of captopril on serum AT-II and colonic epithelial expression of *ACE* and *AT-1R* mRNA in SHRSP-ZF rats

**Figure 2:**
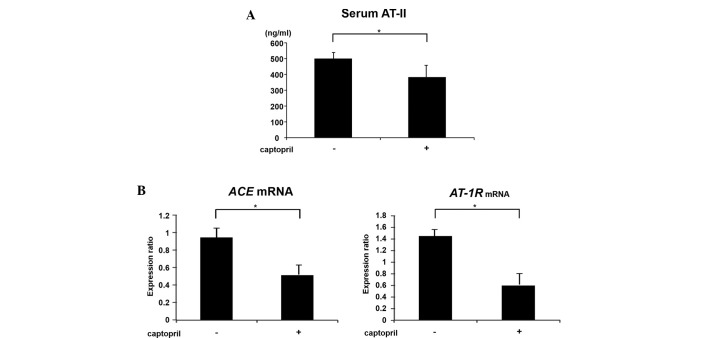
Effects of captopril on serum levels of AT-II and the expression levels of *ACE* and *AT-1R* mRNA in the colonic epithelium of the experimental rats. (A) The serum concentrations of AT-II were measured using enzyme immunoassay and (B) the expression levels of *ACE* and *AT-1R* mRNA in the colonic epithelium were examined by quantitative polymerase chain reaction using specific primers. Data are presented as the mean ± standard deviation. ^*^P<0.01. AT, angiotensin; ACE, aberrant crypt foci; AT-1R, AT-II type 1 receptor.

Hyperactivity of the renin-angiotensin system is implicated in the etiology of Mets and closely correlates with the development and the progression of CRC (16–18). Therefore, the current study investigated the effects of captopril on the serum levels of AT-II and the expression levels of renin-angiotensin system components, including *ACE* and *AT-1R* mRNA in the colonic epithelium. Administration of captopril significantly reduced the levels of serum AT-II (Fig. 2A; P<0.01), and the expression levels of *ACE* and *AT-IR* mRNA in the colonic epithelium were also decreased with captopril treatment (Fig. 2B; P<0.01). These observations indicated that the local level (colonic epithelium), in addition to the systemic level (serum), of renin-angiotensin system activation in diabetic and hypertensive SHRSP-ZF rats was significantly inhibited by captopril.

### Effects of captopril on systemic oxidative stress and colonic epithelial expression of CAT mRNA in SHRSP-ZF rats

**Figure 3:**
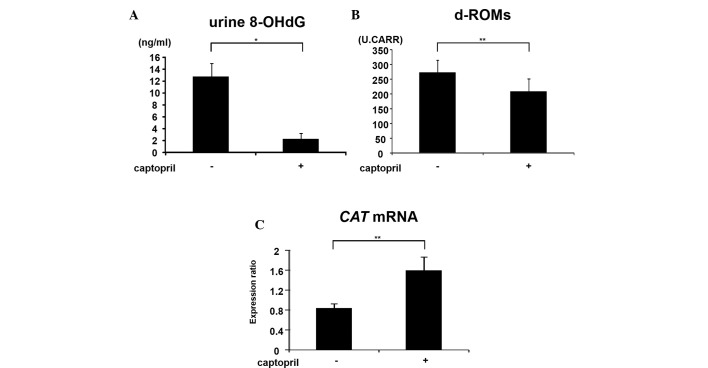
Effects of captopril on urinary levels of 8-OHdG, serum levels of d-ROM and the expression levels of *CAT* mRNA in the colonic epithelium of the experimental rats. (A) Urine 8-OHdG levels were measured by enzyme immunoassay, (B) hydroperoxide levels in the serum were determined by the d-ROM test and (C) the expression levels of *CAT* mRNA in the colonic epithelium were examined by quantitative polymerase chain reaction using specific primers. Data are presented as the mean ± standard deviation. ^*^P<0.001 and ^**^P<0.01. 8-OHdG, 8-hydroxy-2′-deoxyguanosine; d-ROM, derivatives of reactive oxygen metabolites; CAT, catalase.

Oxidative stress is key in Mets-related colorectal tumorigenesis (3,4). Therefore, the current study examined whether captopril administration effects the levels of oxidative stress and antioxidant biomarkers in experimental rats. Captopril administration significantly decreased the levels of urine 8-OHdG (Fig. 3A; P<0.001), a marker of DNA damage induced by oxidative stress and serum d-ROM (Fig. 3B; P<0.01), which reflects serum hydroperoxide levels, in SHRSP-ZF rats. By contrast, in captopril-treated rats, a significant increase was identified in the colonic epithelial expression of *CAT* mRNA, which encodes an antioxidant enzyme (Fig. 3C; P<0.01). These observations suggested that captopril attenuates the systemic and colonic epithelial oxidative stress.

### Effects of captopril on colonic epithelial expression of TNF-α, IL-18, MCP-1, iNOS and VEGF mRNA in SHRSP-ZF rats

**Figure 4:**
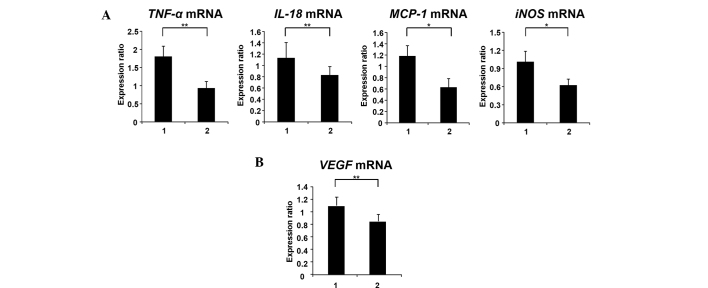
Effects of captopril on the expression levels of *TNF-α*, *IL-18*, *MCP-1*, *iNOS* and *VEGF* mRNA in the colonic epithelium of the experimental rats. The expression levels of these mRNA in the colonic epithelium were examined by quantitative polymerase chain reaction using specific primers. Data are presented as the mean ± standard deviation. ^*^P<0.01 and ^**^P<0.05. *TNF-α*, tumor necrosis factor α; *IL-18*, interleukin 18; *MCP-1*, monocyte chemoattractant protein 1; *iNOS*, inducible nitric oxide synthase; *VEGF*, vascular endothelial growth factor.

Chronic inflammation is associated with Mets and CRC development (3–5). Therefore, the current study examined the effects of captopril on the colonic expression levels of inflammatory mediators in SHRSP-ZF rats. Captopril treatment significantly decreased the colonic epithelial expression of *TNF-α* (P<0.05), *IL-18* (P<0.05), *MCP-1* (P<0.01) and *iNOS* mRNA (P<0.01) in the experimental rats (Fig. 4A). In addition, the expression levels of *VEGF* mRNA, which are upregulated by the AT-II/AT-1R axis (25), were also significantly decreased by captopril treatment (Fig. 4B; P<0.05).

### Effects of captopril on serum levels of glucose, insulin, leptin, NEFA and triglycerides in SHRSP-ZF rats

Insulin resistance and adipokine imbalance are associated with Mets-related colorectal carcinogenesis (3). SHRSP-ZF rats are hyperglycemic, hyperinsulinemic, hyperleptinemic and hypertriglyceridemic compared with their genetic control (10). Therefore, whether captopril treatment alters the serum levels of glucose, insulin, leptin, NEFA and triglycerides in SHRSP-ZF rats was investigated in this study. It was found that captopril treatment did not improve these metabolic parameters in the experimental rats (Table III). The value of QUICKI, a useful index of insulin sensitivity (26), was also not affected by captopril treatment.

## Discussion

Mets and its associated metabolic abnormalities, including diabetes mellitus and hypertension, are significant risk factors for the development of CRC (1–3). Among pathophysiological disorders associated with Mets, in particular hypertension, activation of the renin-angiotensin system is considered to be critical in the early events of colorectal carcinogenesis (10). Dysregulation of the renin-angiotensin system is involved in cancer cell migration and invasion, as well as metastasis in malignant tumors, including CRC (13,16–18). AT-II, which is a main effector peptide in the renin-angiotensin system, has been known to enhance cell proliferation, invasion and survival of CRC cells (27). The gene expression and enzymatic activity of ACE are also increased in human CRC tissues (19). These reports indicated that the activated renin-angiotensin system is mechanistically fundamental in Mets-related colorectal carcinogenesis and, therefore, may be a promising target for the prevention of CRC.

The results of the present study clearly indicated that the administration of captopril, a renin-angiotensin system inhibitor, effectively suppresses the development of AOM-induced colonic preneoplastic lesions in diabetic and hypertensive SHRSP-ZF rats by decreasing the serum levels of AT-II and colonic epithelial expression levels of *ACE* and *AT-1R* mRNA. These observations are consistent with those of a previous study demonstrating that the treatment with an ACE inhibitor and ARB inhibits chemically induced colorectal carcinogenesis in obese and diabetic mice (22). In a human trial, long-term use of an ACE inhibitor also reduced the incidence and size of colorectal adenomas (28). These studies (22,28), together with the results of the present study, markedly suggest that renin-angiotensin system inhibitors, including ACE inhibitors and ARBs, may be useful for the prevention of CRC development in patients with Mets, particularly those with hypertension.

A recent study showed, even without obesity and diabetes, that hypertension *per se* enhances colorectal carcinogenesis and is associated with the elevated levels of oxidative stress (10). Increased levels of AT-II activate the renin-angiotensin system and lead to an increase in oxidative stress (17,29), which is involved in the production of DNA damage and mutations associated with colorectal carcinogenesis (3,30). In this study, captopril administration lowered the blood pressure, decreased the levels of urine 8-OHdG and serum d-ROM, which are implicated in increased oxidative stress (24,31), and increased the expression of CAT, an antioxidant enzyme, thus suppressing the development of AOM-induced ACF in SHRSP-ZF rats, which are subjected to strong oxidative stress (10). These observations are consistent with previous studies (22,32) that have reported the cancer preventive effects of renin-angiotensin system inhibitors via the reduction of oxidative stress.

In addition to oxidative stress, renin-angiotensin system activation is also implicated in the induction of chronic inflammation (16,17,33), which is a key factor for Mets and CRC development (3–5). Activation of AT-1R by AT-II induces a number of molecules that participate in inflammatory responses (16,17,34). AT-II also induces the expression of iNOS, an inflammatory marker, along with 8-OHdG in cancer cells through the activation of AT-1R (32), suggesting a cross-link between renin-angiotensin system-related inflammation and oxidative stress in cancer tissue. In addition, AT-II stimulates the expression of VEGF through the activation of AT-1R and the induction of chronic inflammation (25). In the present study, captopril administration decreased the expression levels of *TNF-α*, *IL-18*, *MCP-1*, *iNOS* and *VEGF* mRNA in the colonic epithelium of AOM-treated SHRSP-ZF rats. Therefore, in addition to the reduction of oxidative stress, the chemopreventive effect of captopril on Mets-related colorectal carcinogenesis is most likely associated with the attenuation of systemic inflammation.

Pathological conditions implicated in Mets, such as insulin resistance, hyperleptinemia and dyslipidemia, may be critical therapeutic targets in the prevention of obesity- and diabetes-related colorectal carcinogenesis (4,6–9). However, in the present study, captopril treatment did not improve these metabolic disorders. These observations, together with the results of a recent study (10), may suggest that the renin-angiotensin system is a promising target for preventing early-phase colorectal carcinogenesis associated with Mets, in particular, hypertension. To confirm this hypothesis, experiments of longer duration are required to determine whether renin-angiotensin system inhibitors actually suppress Mets-related CRC development by suppressing the activation of the system. In addition, the possibility of combination chemoprevention using renin-angiotensin system inhibitors and specific drugs for Mets (such as antidiabetic drugs, which improve insulin resistance) for preventing Mets-related colorectal carcinogenesis must also be explored.

In conclusion, targeting Mets-related metabolic abnormalities, particularly the activation of the renin-angiotensin system and subsequent induction of oxidative stress and inflammation, may be an effective strategy to prevent the development of CRC in patients with Mets. Renin-angiotensin system inhibitors, including ACE inhibitors, appear to be potentially effective and viable candidates for this purpose since these agents reduce oxidative stress while also attenuating chronic inflammation.

## Figures and Tables

**Table I:** Primer sequences.

Target gene	Direction	Primer sequence (5′-3′)
*TNF-α*	Forward	AACACACGAGACGCTGAAGT
	Reverse	TCCAGTGAGTTCCGAAAGCC
*IL-18*	Forward	ACAGCCAACGAATCCCAGAC
	Reverse	ATAGGGTCACAGCCAGTCCT
*MCP-1*	Forward	TGGGCCTGTTGTTCACAGTT
	Reverse	ACCTGCTGCTGGTGATTCTC
*iNOS*	Forward	GTGGTGACAAGCACATTTGG
	Reverse	GGCTGGACTTTTCACTCTGC
*ACE*	Forward	CTTGACCCTGGATTGCAGCC
	Reverse	GTTTCGTGAGGAAGCCAGGA
*AT-1R*	Forward	TCGTGGCTTGAGTCCTGTTC
	Reverse	CGCGCACACTGTGATATTGG
*VEGF*	Forward	TCCACCGTGTATGCCTTCTCC
	Reverse	CCTGCTGTATCTGCGCACTGGA
*CAT*	Forward	GAGGCAGTGTACTGCAAGTTCC
	Reverse	GGGACAGTTCACAGGTATCTGC
*PCNA*	Forward	AAGACCTCGCTCCCCTTACA
	Reverse	ATCAGGCGTGCCTCAAACAT
*GAPDH*	Forward	CCTTCATTGACCTCAACTACATGGT
	Reverse	TCATTGTCATACCAGGAAATGAGCT

TNF-α, tumor necrosis factor; IL-18, interleukin-18; MCP-1, monocyte chemoattractant protein-1; iNOS, inducible nitric oxide synthase; ACE, angiotensin-converting enzyme; AT, angiotensin; AT-1R, AT-II type 1 receptor; VEGF, vascular endothelial growth factor; CAT, catalase; PCNA, proliferating cell nuclear antigen; GAPDH, glyceraldehyde-3-phosphate dehydrogenase.

**Table II:** Body weights, adipose tissue weights and blood pressure of the experimental rats.

Group	n	Treatment	Body weight, g	Relative adipose tissue weight, g/100 g body weight^a^	Blood pressure, mmHg	

					Systolic	Diastolic
1	10	AOM	270.7±20.1^b^	1.67±0.16	170±13.1	130±8.6
2	10	AOM + captopril	261.4±4.1	1.97±0.24^c^	146±15.4^c^	112±14.2^c^

a White adipose tissue of the periorchis and retroperitoneum.

b Mean ± standard deviation.

c P<0.05 vs. group 1, as determined by Student’s t-test.

AOM, azoxymethane.

**Table III:** Serum parameters of the experimental rats.

Group	n	Glucose, mg/dl	Insulin, μIU/ml	Quicki	Leptin, pg/ml	NEFA, mEq/ml	Triglyceride, mg/dl
1	10	120.0±14.2^a^	25.6±9.0	0.29±0.01	102.7±30.6	537.9±30.0	257.1±79.4
2	10	118.5±15.4	25.5±7.2	0.28±0.02	101.2±27.7	555.0±27.8	234.7±64.5

a Mean ± stand deviation.

NEFA, non-esterified fatty acid.
